# Temperate bacteriophages infecting the mucin-degrading bacterium *Ruminococcus gnavus* from the human gut

**DOI:** 10.1080/19490976.2023.2194794

**Published:** 2023-03-30

**Authors:** Colin Buttimer, Ekaterina V. Khokhlova, Lisa Stein, Cara M. Hueston, Bianca Govi, Lorraine A. Draper, R. Paul Ross, Andrey N. Shkoporov, Colin Hill

**Affiliations:** a APC Microbiome Ireland and School of Microbiology, University College, Cork, Ireland; b School of Microbiology, University College Cork, Cork, Ireland

**Keywords:** *Ruminococcus gnavus*, bacteriophage, mouse trial, inflammatory bowel disease

## Abstract

*Ruminococcus gnavus* is a prevalent gut microbe reported to occur in higher abundance among individuals with inflammatory bowel disease (IBD). This study reports the isolation and characterization of six bacteriophages (phages) isolated from human fecal material and environmental samples that infect this species. Isolated phages have a siphovirus morphology, with genomes ranging between 36.5 and 37.8 kbp. Genome analysis indicates that the phages have a temperate lifestyle, which was confirmed by their ability to form lysogens on their host bacterial species. In contrast to the finding that phages lyse their host in liquid medium, results from a mouse trial indicate these phages can co-exist with the host bacterium in the gut without causing a significant reduction of *R. gnavus*. The bacterial counts in the feces of phage-treated mice did not significantly differ in the presence of phage. Furthermore, analysis of publicly available gut virome sequence data indicates a high abundance of these phages among individuals suffering from IBD. This work provides the first insight into how phages interact with *R. gnavus* in the human gut microbiome.

## 1. Introduction

The human gut microbiome is composed of a dynamic community of numerous microorganisms that engage in different types of symbiotic interactions (mutualism, commensalism, and parasitism) with the metazoan host. Mutualistic microbial symbionts provide a whole array of crucial functions to the human guts, such as digesting complex dietary carbohydrates and synthesizing bioactive molecules (vitamins, amino and fatty acids).^1,2^ They modulate the human immune system directly (cellular components) or indirectly by producing immunomodulatory substances.^3,4^ They can also act as a barrier against invading pathogenic bacteria.^5^ In the healthy human gut, the microbial community exists in a steady state, resulting in long-term stability of its taxonomic and functional composition.^6^ Insults, such as antibiotic treatments or inflammation, cause the gut microbial community to depart from that steady state, and adopt a new (meta)stable “dysbiotic” state.^7^ Furthermore, altered gut microbiomes have been observed among people afflicted with inflammatory bowel diseases (IBD), such as Crohn’s disease (CD) and ulcerative colitis (UC).^8^

*Ruminococcus gnavus* is a gram-positive anaerobe that is widely prevalent in the gut microbiome of individuals. The most recent literature places the bacterium within the family *Lachnospiraceae*, ^9^ and to avoid confusion with other ruminococci classified under family *Oscillospiraceae* a renaming of *R. gnavus* as *Mediterraneibacter gnavus* has been proposed but not validly published.^10^ The bacterium is 1 of the 57 species that was detected in the feces of more than 50% of 124 European individuals.^11^ The widespread prevalence of *R. gnavus* in the gut of individuals can likely be explained by its ability to utilize human mucin as a nutrient source, as observed with *R. gnavus* strain ATCC 29149, which can produce an intramolecular trans-sialidase encoded by the *RgNanH* gene producing 2,7-anhydro-Neu5Ac selectively from α2–3-linked sialic acid substrates.^12^ This makes the enzyme distinct from hydrolytic sialidases found in other mucin-degrading bacteria, such as *Bacteroides thetaiotaomicron or Akkermansia muciniphila* that produce free sialic acid (Neu5Ac).^12^

In addition, *R. gnavus* has evolved to survive and thrive in competitive microbial communities through the production of bacteriocins. For example, *R. gnavus* E1 was shown to produce the antimicrobial sactipeptide Ruminococcin C *in vivo* during the colonization of the digestive tract of rats.^13^ Another aspect of *R. gnavus* adaptation to the human gut is its involvement in host metabolic pathways, including bile acid metabolism with the production of iso-bile acids.^14^

*R. gnavus* is particularly prevalent among individuals with IBD, especially those suffering from CD.^15–17^ Moreover, blooms of this bacterium among such individuals can be temporal and often correlate with disease flare. The relative abundance of *R. gnavus* in CD patients can reach a maximum of 69.5% compared to less than 1% in healthy individuals.^16,18^ Production of capsular polysaccharides (CPS) has been implicated in playing a role in the onset of IBD, where strains of *R. gnavus* that produce CPS can exist symbiotically with the host immune system. In contrast, strains that do not produce CPS can elicit an inflammatory immune response.^19^ Another explanation given for the bacterium’s possible role in the generation of inflammation associated with CD is its production of the polysaccharide glucorhamnan that can induce the toll-like receptor 4 (TLR4)-dependent secretion of a pro-inflammatory cytokine tumor necrosis factor-α (TNFα) in dendritic cells.^20^ The bacterium is also associated with other inflammatory non-IBD conditions, such as spondylarthritis.^21^

Bacteriophages (phages) are viruses that specifically infect bacteria. Phage numbers are estimated to range between 1:1 and 1:100 compared to bacteria in the human gut.^22^ They often display a narrow host range, with their infection typically limited to specific strains within a bacterial species. There is growing interest in their development as alternatives to combat bacterial diseases and tools for microbial gut engineering.^23,24^

Most phages infect their bacterial host through either lytic or lysogenic life cycles. Lytic phages kill their bacterial host through cell lysis, whereas lysogenic phages integrate into the bacterial host genome. Metagenomic studies of the human gut virome show a rich diversity of phages associated with this ecological niche, where phages found within the gut virome are stable over time but highly individual-specific.^22,25,26^ Individuals with IBD are enriched in temperate phage-related sequences in the gut virome as compared with healthy individuals.^27^

To our knowledge, phages infecting *R. gnavus* have not yet been documented. The current study describes the biological characterization and genomic and phylogenetic analyses of six phages related at the genus to subfamily level infecting *R. gnavus*, obtained from human fecal and environmental samples.

## 2. Materials and methods

### 2.1. Bacterial strains and culture conditions

*R. gnavus* strains were sourced from the Japan Collection of Microorganisms (JCM 6515^T^ = ATCC 29,149^T^) and the Culture Collection University of Gothenburg (CCUG 52,279, CCUG 51,289, CCUG 54,531, CCUG 43,222, CCUG 49,994, CCUG 57,161, CCUG 57,208, and CCUG 57,137). Upon random screening with 16S rRNA species-specific PCR primers, the *R. gnavus* strain PS/160 was obtained out of a collection of strict anaerobes isolated from pooled human feces (unpublished data). All *R. gnavus* strains were propagated using Anaerobe basal broth (ABB, Oxoid – ThermoFisher). All manipulations were carried out at 37°C in strict anaerobic conditions (Type A vinyl anaerobic chamber, Coy Labs).

### 2.2. Fecal sample collection and the isolation and propagation of phages

Fecal samples used in the present study were randomly selected from a subset of a larger IBD study cohort.^28^ A total of 33 samples were selected from healthy volunteers and 45 samples from IBD-affected individuals. Manure/slurry tank samples were collected from several locations around a single multi-species farm in Co. Tipperary, Ireland. Human feces and farm samples were split into 1 g aliquots and resuspended in 10 mL of SM buffer (50 mM Tris-HCl pH [7.5], 100 mM NaCl, 8 mM MgSO_4_). These suspensions were centrifuged, with the resulting supernatants being filtered twice through 0.45 µm pore membrane filters. Then, 2 mL of these filtrates were mixed with 2 mL of 2× ABB soft agar (final agar concentration 0.4% w/v) and 0.5 mL of an overnight culture of *R. gnavus* JCM 6515^T^ to create an overlay spread on ABB 1.5% w/v agar plates. Plaque formation was assessed after 16–24 h of incubation. Individual plaques were excised and soaked in 100 µL of SM buffer and incubated for 2–3 h with occasional shaking to separate phages from the top agar. The resulting phage preparations were filtered through 0.45 µm pore spin filters. The resulting phage preparation was replated in the same manner as previously described, with this process repeated twice more to produce pure phage stocks.

Two to three rounds of plate propagation were performed to create high-titer phage lysates, followed by two to three rounds of liquid propagation. Briefly, top agar from three plates with near confluent lysis (~10^4^ plaques per plate) was collected in a 15 mL falcon tube, vigorously shaken for 2–3 h, and centrifuged to pellet debris. Obtained supernatants were filtered through 0.45 µm pore membrane filters and used for the subsequent round of propagation. A volume of 0.5–1 mL of phage lysate (or at MOI = 1) was added to 10 mL of exponentially growing host strain *R. gnavus* JCM 6515^T^ in ABB broth (OD_600_ = 0.2) and incubated until clearing was observed. Lysates were centrifuged into pellet debris with the supernatant filter sterilized. Phage lysates were stored at 4°C for further experiments.

### 2.3. The efficiency of lysogeny and host range analysis

For the efficiency of the lysogeny test, 200 µL of high-titer (>10^9^ PFU/mL) phage lysates were spread onto ABB agar plates. A volume of 100 µL of serial dilutions of overnight cultures *R. gnavus* JCM 6515^T^ were used to inoculate phage-covered and control agar plates. The efficiency of lysogeny was calculated after 48 h of incubation for a percentage of colonies grown on phage-seeded plates relative to the total bacterial number on unseeded/control plates. Ten randomly selected colonies (for each phage) from phage-covered plates were transferred onto ABB agar plates. After three subsequent rounds of streaking, colonies were checked for the presence of prophages using the following primers: phiPS6-F1, R1; phiRg507T2/2-F1, R1; phiRg507T2/3-F1, R1 (Supplementary information 2, Table S1).

The host range of the phages was determined by spotting 10 µL of phage lysates onto a bacterial lawn prepared by the double agar overlay method. Briefly,: 5 mL of molten soft ABB agar (0.4% w/v) was mixed with 0.3 mL of overnight bacterial culture and spread onto ABB agar plates. Phage lysates were spotted after the solidification of the top layer. The formation of the spots was assessed after 16–24 h of incubation.

### 2.4. Phage kill-curve assays

For the phage killing curve assays, a culture of *R. gnavus* JCM 6515^T^ was grown from ABB to OD_600_ of 0.3 (10^8^ CFU/mL). A volume of 90 µL of cell suspension was dispensed into the wells of flat-bottom 96-well plates. Ten microliters of phage lysate (phiRgPS6, phiRg507T2/2, phiRg507T2/3, and phiRg519T2) or phage mixtures in SM buffer were added to the wells at different MOIs − 1, 0.1, 0.01 and 0.001. Negative control wells were inoculated with SM buffer only. Plates were sealed and immediately transferred to the plate reader. Measurements were taken to monitor OD_595_ changes for 24 h at 30 min intervals of 5 s shaking before readings. Experiments were performed twice in technical duplicates for each phage or phage mixture. The outcome of these experiments was visualized with the ggplots2 package in R.

### 2.5. Plaque morphology and electron microscopy

Briefly: 60 mL of high-titer phage lysates (>10^9^ PFU/mL) were spun down in a ultra-centrifuge using an F65L-6×13.5 rotor (ThermoFisher) at 120,000 × g for 3 h. The total phage pellet was resuspended in 5 mL of SM buffer and subsequently purified by a cesium chloride step gradient (5 M/3 M CsCl solutions) with ultra-centrifugation at 105,000 × g for 2.5 h. The phage band was collected by a syringe and subjected to three rounds of buffer exchange by 10-fold dilution with SM buffer and concentrated to the initial volume using Amicon Centrifugal Filter Units MWCO 10 KDa (Millipore). Five microliters of purified phage sample were applied onto Formvar/Carbon 200 Mesh, Cu grids (Electron Microscopy Sciences), negatively stained with 0.5% w/v uranyl acetate and examined by a Tecnai G2 12 BioTWIN transmission electron microscope at UCD Conway Imaging Core Facility (University College Dublin, Dublin, Ireland).

### 2.6. *In vivo* mouse study

Ten germ-free C57BL/6 male mice (8 weeks old, bred in-house) were separated into two groups: phage administration group (*n* = 5) and SM-buffer administration group (control group; *n* = 5). Each group of mice was housed in two cages, with two and three mice, respectively. Mice were inoculated by oral gavage with 0.2 mL *R. gnavus* JCM 6515^T^ cells in PBS (2 × 10^9^ CFU) on days 1, 2, and 3 of the experiment. On days 11, 12, and 14, all mice were inoculated by oral gavage with 0.2 mL: of a phage suspension (phiRg507T2/2, phiRg507T2/3, and phiRgPS6) in SM buffer at 10^10^ PFU for the phage treatment group or 0.2 mL SM buffer for the control group. On days three, seven, 11–23 (once every 2 days), and day 26 mice were placed in new cages and approximately 10 fresh fecal pellets were collected from each cage. Pellets were processed on the same day as collection to ensure the optimal viability of *R. gnavus*. On day 26, all mice were sacrificed.

To enumerate *R. gnavus* in mouse feces, feces was resuspended in PBS containing 0.3 mg/mL L-cysteine HCl, 0.3 mg/mL Na-thioglycolate, 1 mg/mL DTT. This underwent a ten-fold serial dilution and was plated onto ABB agar and incubated in anaerobic conditions at 37°C. For phage enumeration, the 10^−2^ dilution used for bacteria enumeration was filtered-sterilized and used for a ten-fold serial dilution that was subsequently spotted onto an overlay with *R. gnavus* JCM 6515^T^ as previously described. GraphPad Prism (v8) was used to determine the normality of data using the Shapiro–Wilk test. The unpaired t-test was utilized to compare treatment groups.

On day 23, enumerated *R. gnavus* isolates were checked for prophages using the following primer pairs: phiRgPS6-F1/R1; phiRg507T2/2-F1/R1; phiRg507T2/3-F1/R1 (Supplementary information 2, Table S1).

### 2.7. DNA isolation and genome sequencing of *Ruminococcus* phages

Genomic DNA extraction was conducted as previously described.^29^ Briefly, phage PEG/NaCl precipitates were collected by centrifugation at 5200 × g for 20 min at 4°C. Pellets were resuspended in 400 µL of SM buffer and extracted by gentle shaking with an equal volume of chloroform followed by centrifugation at 2500 × g for 5 min. The aqueous phase (~360 µL) was aspirated into clean Eppendorf tubes and treated with 8 U of TURBO DNase (ThermoFisher) and 20 U of RNase I (ThermoFisher) in the presence of 1 mM CaCl_2_ and 5 mM MgCl_2_ at 37°C for 1 h before inactivating enzymes at 70°C for 10 min. This was followed by a proteinase K (40 µg) treatment in the presence of 0.5% w/v SDS for 20 min at 56°C. Viral particles were then lysed by the addition of 100 µL of Phage Lysis Buffer (4.5 M guanidinium isothiocyanate, 44 mM sodium citrate [pH 7.0], 0.88% w/v sarkosyl and 0.72% v/v 2-mercaptoethanol) at 65°C for 10 min. Lysates were extracted twice with an equal volume of phenol/chloroform/isoamyl alcohol 25:24:1 (ThermoFisher) and subjected to a final round of DNA purification using DNeasy Blood & Tissue Kit (Qiagen). DNA was then quantified using Qubit dsDNA HS Assay Kit and subjected to shotgun library preparation using TruSeq Nano DNA or Nextera XT kits (Illumina), followed by sequencing on the Illumina HiSeq 2500 platform (GATC Biotech AG). Illumina reads were trimmed and filtered using Cutadapt v.2.4 and Trimmomatic v.0.36 as described before,^30–32^ then assembled with SPAdes v3.10.0.^33^

### 2.8. DNA isolation and genome sequencing of *R. gnavus*

Briefly, genomic DNA extraction was performed on overnight cultures of *R. gnavus* in ABB using the GenElute kit (Merck), following the manufacturer’s instructions. The Qubit dsDNA HS Assay Kit and Qubit 2.0 Fluorometer (Life Technologies) were used to determine the DNA concentration. The Nextera DNA Flex Library Preparation Kit (Illumina) was used to generate DNA libraries, followed by sequencing on the Illumina NovaSeq platform (Illumina) that was outsourced to AZENTA Life Sciences. Oxford Nanopore sequencing using the Rapid Barcoding Kit (SQK-RBK004; Oxford Nanopore) was performed on the MinION Mk1B platform using MinKNOW software for selected *R. gnavus* strains. Hybrid assembly of Illumina (trimmed and filtered as above) and Oxford Nanopore reads trimmed and filtered using NanoFilt v2.8.0 was conducted using SPAdes for JCM 6515^T^, and using Unicycler v0.4.8 for all other strains.^34–36^

### 2.9. Bioinformatic analysis

Phage genomes were initially annotated with RASTtk.^37^ Open reading frames (ORFs) were further investigated using BLASTp using the nr (November 2021) and Phaster (December 2020) databases, Interproscan v5.57–90.0 and HHpred. ARAGORN was used to identify tRNA genes.^38–42^ Phage genome nucleotide similarity was calculated using VIRIDIC and orthologous genes were discovered by Coregenes 5.0.^43,44^ Phylogenetic analysis based on the phage proteome was performed with VIPtree and VICTOR.^45,46^ Phylogenetic trees were edited with iTOL.^47^ Gegenees v3.1.0 was used to calculate phage proteome homology.^48^ Pangenome analysis of proteomes was conducted with Proteinortho v6.1.2 [command: proteinortho *.faa -identity = 30 -cov = 70 -singles].^49^ The resulting.tsv presence-absence table of orthologous groups (OGs) was inspected and visualized using the stats, reshape, gridextra, tidyverse and ggplots2 packages in R. For the presence-absence map of OGs among phages, the proteinothro.tsv file of identified OGs was correlated to protein annotation of analyzed phages. Bacterial genomes were annotated with Prokka v1.14.6, using the Phaster and pVOGs databases for identification of prophage gene products.^41,50,51^ Pyani v0.2.7 was used for the calculation of average nucleotide identity between bacterial genomes.^52^ Identified prophages in the genomes of *R. gnavus* were manually reviewed using the Artemis Comparison Tool (ACT) v18.1.0 or Mauve v10.^53,54^ Minced v0.4.2 (ctSkennerton/minced) was used for detection of CRISPR spacer sequences and BLASTn (-task BLASTn-short) was used to identify protospacers among phage genomes, possessing no more than 3 misaligned bases.

### 2.10. Analysis of the prevalence of *Ruminococcus* phages in the gut microbiome

The human fecal shotgun virome and 16S rRNA amplicon sequencing data previously reported by Norman *et al*. were accessed through BioProject PRJEB7772.^55^ Briefly, sequencing reads were processed with Trimmomatic v0.36 for trimming and filtering. Virome reads were aligned to phage genomes using Bowtie v2.1.0 with counts of aligned reads extracted from alignment data with SAMtools v0.1.19 as previously described.^31,56–58^ For 16S rRNA amplicon reads, counts were generated using BBmap v39.01 on reads aligning with 97% identity [idfilter = 0.97] to 16S rRNA sequences of *Ruminococcus gnavus* obtained from the Silva 138.1 prokaryotic SSU database.^59^ Counts of aligned reads were extracted with SAMtools. Boxplots were generated with ggplot2 in R.

### 2.11. Data availability

Genomes of bacteria and phages described in this study are available at GenBank under accession numbers given in Table 1

**Table 1.** GenBank accession numbers for genomes of phages and bacteria sequenced in this study.

Genomes		accession number
*Ruminococcus* phage	phiRg507T2/2	MT980836
	phiRg507T2/3	MT980837
	phiRg519T2	MT980838
	phiRgPS6	MT980839
	phiRgIBDN1	MT980840
	phiRM10	MT980841
*R. gnavus bacterial strains*	JCM 6515^T^ = ATCC 29149T	CP043051
	JCM 6515^T^ (phiRgPS6) clone 1–1	CP111084
	JCM 6515^T^ (phiRgPS6) clone 2–1	CP111086
	CCUG 52,279	JAPRBD000000000
	CCUG 51,289	JAPRBC000000000
	CCUG 54,531	JAPRBB000000000
	CCUG 43,222	JAPRBA000000000
	CCUG 49,994	JAPRAY000000000
	CCUG 57,161	JAPRAX000000000
	CCUG 57,208	JAPRAW000000000
	CCUG 57,137	JAPRAV000000000
	PS/160	JAPRAU000000000

2.12.

## 3. Results

### 3.1. Isolation of phages against *R. gnavus* JCM 6515^T^

Seventy-eight fecal samples from human donors (healthy and those suffering from IBD) and 23 samples of animal and environmental origin (multi-species farm) were subjected to phage screening against *R. gnavus* JCM 6515^T^ (= ATCC 29,149^T^). Twelve samples (a pooled fecal sample, stool from eight individuals with IBD, and three farm animal samples) produced plaques. These plaques were observed to be small in size (1–3 mm in diameter) and clear. *Ruminococcus* phage phiRg507T2/3 was the only isolate to produce plaques surrounded by halos (Supplementary information 1, Figure S1). Plaques from the stool of a healthy individual and those suffering with IBD and the three farm samples were purified through triple passage with isolation of a single plaque on each step. Phages from sample 507T2 yielded two distinct types of plaque morphologies (medium-sized with halos or those pinprick in size without halos), which were purified separately. Ultimately, six phage isolates were selected, which were denoted as phiRgPS6 (pooled fecal sample isolate), phiRg507T2/2, phiRg507T2/3, phiRg519T2, phiRgIBDN1 (IBD isolates), and phiRgRM10 (farm isolate).

### 3.2. Biological characterization of the phages

**Figure 1.**
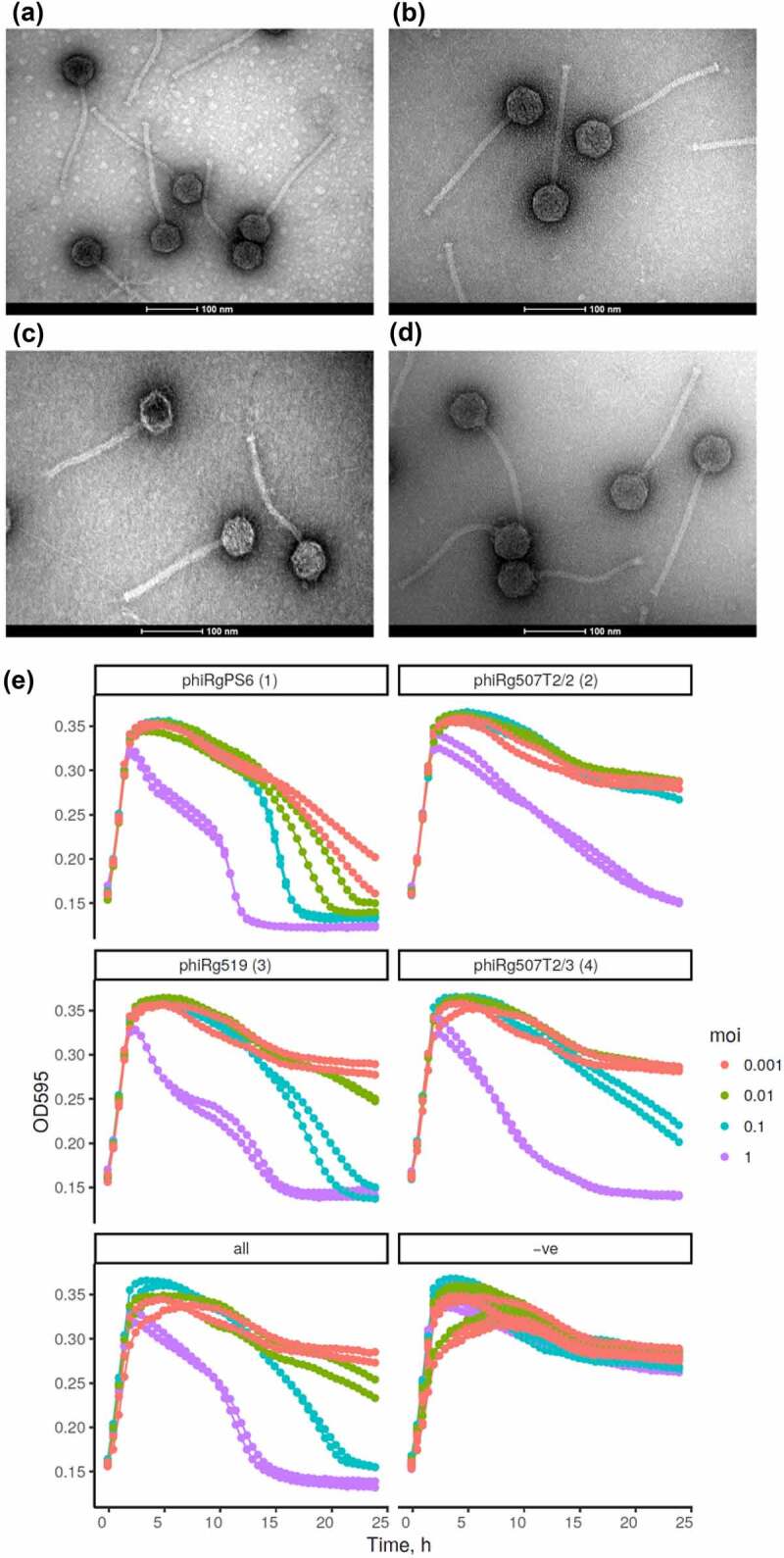
Transmission electron micrographs and phage-host kill curves. The micrographs of *Ruminococcus gnavus* phages (a) phiRg507t2/2, (b) phiRg507t2/3, and (c) phiRgps6, (d) phiRg519. The scale bar represents 100 nm. (e) Phage kill-curve assays of *Ruminococcus gnavus* JCM 6515^T^ in response to infection by *Ruminococcus* phages phiRgps6, phiRg507t2/2, phiRg507t2/3, phiRg519t2 and a phage cocktail of these four phages at varying MOIs from 1×10^−3^ to 1, compared to a negative control (−ve) of untreated culture of *R. gnavus* JCM 6515^T^.

Transmission electron microscopy of phiRgPS6, phiRg507T2/2 and phiRg507T2/3 revealed virions with a typical siphovirus morphology with icosahedral heads (55–61 nm in diameter), with long flexible non-contractile tails (195–204 nm) possessing very discreet baseplates (Figure 1a–d and Supplementary information 2, Table S2).

The narrow host range of these phages was confirmed by spotting phage lysate on overlays seeded with eight different strains of *R. gnavus* obtained from the CCUG collection and strain PS/160, which was isolated during this study. All but two phages had lytic activity limited to a single host strain JCM 6515^T^. Phages phiRgPS6 and phiRgIBDN1 additionally produced lysis on *R. gnavus* CCUG 57137 (Supplementary information 2, Table S3).

Kill curve assays were performed to evaluate the lytic activity of phages against *R. gnavus* in liquid medium. Exponentially growing cultures of JCM 6515^T^ were inoculated with phiRgPS6, phiRg507T2/2, phiRg507T2/3 and phiRg519T2 and a combination of these four phages at four different multiplicities of infection (MOI = 1; 0.1; 0.01; and 0.001). It was found that each phage on its own and a combination of four could cause effective lysis of the bacterial host after 2 h of growth when added at MOI of 1 (Figure 1e). Moreover, bacterial growth remained inhibited for 10–15 h after the initial lysis event. One of the tested phages, phiRgPS6, was able to suppress the host at all tested MOIs.

These phages were also evaluated for their ability to lysogenize their host. Plating of strain JCM 6515^T^ onto agar seeded with a high titer of *Ruminococcus* phage lysate resulted in isolated colonies that were positive in phage-specific PCR. The efficiency of lysogeny (frequency of formation of lysogens) among the tested colonies was estimated at 35% for phiRgPS6 and 46% for either phage phiRg507T2/2 or phage phiRg507T2/3.

### 3.3. Genome analysis of *Ruminococcus* phages and *R. gnavus* strains

**Figure 2.**
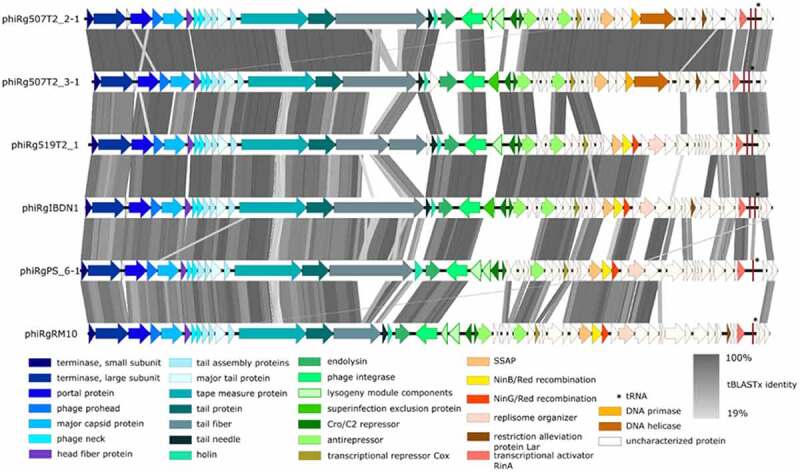
Linear genome map of *Ruminococcus* phages. Comparison of the genomes of *Ruminococcus* phages isolated in this study employing BLASTn and visualisation with Easyfig. The genome maps display arrows indicating the locations and orientation of ORFs among different phage genomes. Arrows have been colour-coded describing their predicted roles (see key), and shading between the genome maps indicates the level of identity.

The genomes obtained for the *Ruminococcus* phages ranged between 36,510 and 37,780 bp (coverage > 1000×) with a GC content of 41–42%. A comparison of nucleotide similarity between genomes showed that all phage isolates were homologous at the nucleotide level. Following the International Committee on Taxonomy of Viruses (ICTV) guidelines,^60^ a shared nucleotide similarity of less than 95% between phages phiRgPS6, phiRg507T2/2, phiRg507T2/3, phiRg519T2, phiRgIBDN1 and phiRgRM10 allows each to be defined as representing a novel species (Supplementary information 1, Figure S2). Additionally, shared nucleotide between genomes exceeds 55%, with gene synteny conserved between the genomes (Figure 2).

The number of open reading frames (ORFs) found on the genomes of these phages ranged from 54 to 59, with each phage also possessing either one or two tRNA genes (Supplementary information 2, Table S4). To examine the shared proteome of the six phages, we conducted a pangenome analysis of their proteins (identity = 30%, coverage = 70%). These proteins could be placed into 117 orthologous groups (OGs), with the shared proteins (core proteome) among these genomes comprising of 20 OGs (17% of the total). Each phage was found to possess between 2 and 12 unique proteins not shared among other isolates (Supplementary information 2, Figure S3). Only 54% of OGs could be given functional assignments, with overlap seen among numerous OGs annotated with similar functions. Our annotation efforts could place OGs into six major functional categories: lysogeny, virion assembly, host lysis, DNA-related, transcriptional regulation and accessory (Supplementary information 1. Figure S4).

OGs associated with virion structure and assembly were found to be the most conserved among the six phages, with 14 of the 20 OGs universally shared among these phages related to this function. These contained proteins implicated in the formation of the capsid (major capsid & prohead), tail (major tail, tape measure and tail assembly chaperone) and their connection (head-tail connector), as well as those involved in genomic DNA encapsulation (portal protein, large and small terminase). However, proteins implicated to be located in the tail and head fiber structures were found not to be conserved among phages.

All phages share a similar tyrosine integrase (InterPro IPR013762). However, a diverse range of proteins containing the Cro/C1-type HTH domain (IPR001387) is distributed among the phages, being placed among five different OGs. Proteins with this domain are expected to be implicated in the transcriptional repression or activation of prophages entering the lytic life cycle. Seven other OGs involved in transcriptional regulation were also identified.

The greatest diversity was found among the 16 OGs in the DNA replication-related category, with no OGs being shared among all six phages. OGs in this category were annotated as endonuclease, recombinase, single-stranded DNA binding protein, double-strand repair protein, replisome organizer or proteins found to possess a DNA binding or zinc finger domain.

All phages possess a predicted endolysin with an N-acetylmuramoyl-L-alanine amidase activity (IPR002508, IPR002502) and a holin, with proteins of each type placed into two different OGs.

Seven OGs were classified as having an accessory role, with proteins in this category not shared universally among phages. These OGs include gene products implicated in overcoming the host restriction-modification systems, such as DNA methyltransferase (IPR001091) for DNA methylation and the Lar family restriction alleviation protein (PF14354), which provides a countermeasure to restriction enzymes targeting foreign DNA. This group also includes proteins that promote resistance to incoming phage infection, such as superinfection exclusion protein (IPR011434) and siphovirus gp157 (IPR008840).

We sequenced the genomes of the 10 *R. gnavus* strains utilized in this study to determine if we could identify factors potentially implicated in the resistance of hosts to the *Ruminococcus* phages. The host strain *R. gnavus* JCM 6515^T^ was sequenced using Illumina and Oxford Nanopore platforms, generating short and long sequence reads, respectively. This allowed the assembly of a complete circular genome of 3.6 Mbp in size (coverage 88.2×). The remaining nine isolates were sequenced using short reads, resulting in high-quality draft genomes (coverage > 400×) with a low number of contigs (54 [42–102]) and a high N50 (161,630 [131,253–245,696]) (median [range]), with low rates of duplication marker genes (Supplementary information 2, Table S5). The average total length of these 10 assemblies was 3.12 ± 0.19 Mbp (medium ± SD), sharing an average nucleotide identity (ANI) of > 97% with the genome of *R. gnavus* JCM 6515^T^.

Of the isolates sequenced, CRISPR arrays were detected among the genomes of CCUG 51289, CCUG 43437 and the host strain JCM 6515^T^ downstream of several Cas proteins associated with a type I-C system. These CRISPR arrays contain 22 ± 5 spacers with a length of 34 ± 1 bp (mean ± SD). A collective total of 68 spacers were identified, with 15 aligning to the *Ruminococcus* phage genomes with no more than three mismatched bases (Supplementary information 2, Table S6). The three bacterial strains were found to have spacers targeting all six phages. Interestingly, JCM 6515^T^ possesses spacers that perfectly match the target protospacer associated with the genomes of phiRg507T2/2 and phiRg507T2/3 while being sensitive to both phages.

We were also curious to learn if the *R. gnavus* strains used in this study possess similar prophage elements with lysogenic repressors that could impact infection by the six *Ruminococcus* phages. Pangenome analysis (identity = 30%, coverage = 70%) with these *Rumnoococcus* phages and 10 *R. gnavus* strains did not reveal the presence of prophage elements that shared conserved proteins (large terminase, capsid & portal protein) found among these phages. Furthermore, no gene products were found to be universally shared between phages and these bacterial strains.

### 3.4. Phylogenetic analysis of *Ruminococcus* phages

These *Ruminococccus* phages do not fall into any phage genus currently recognized by the ICTV. Analysis of these phage genomes using BLASTn with the nt database does not identify any cultured phages with genomes that share significant nucleotide homology with those isolated in this study. However, examples of metagenome-assembled genomes (MAGs) of phages originating from the human microbiome could be identified with genus-level nucleotide similarity (Supplementary information 1, Figure S5). This observation indicates that similar *Ruminococcus* phages are associated with the human microbiome. Additionally, it was possible to identify genomes of *R. gnavus* with prophage elements that share nucleotide similarity of up to 68% with the *Ruminococccus* phages (Supplementary information 1, Figure S5).

**Figure 3.**
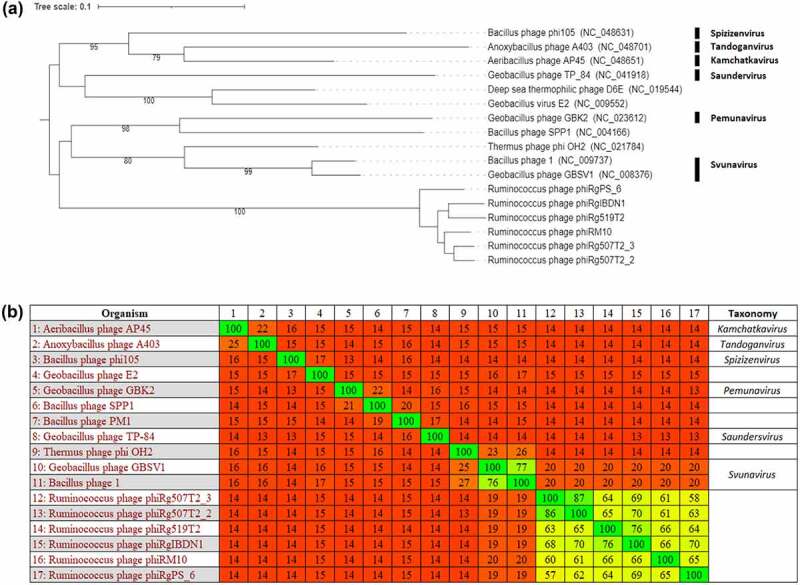
Phylogenetic analysis of *Ruminococcus* phages. (A) VICTOR-generated phylogenomic Genome-BLAST Distance Phylogeny (GBDP) tree inferred using the formula D4 and yielding average support of 67%. (B) a tBLASTx heatmap generated using Gegenees with accurate parameters – fragment length: 200 bp; and step size: 100 bp; threshold: 5%. The phylogram and heatmap includes this study’s *Ruminococcus* phages and those that share an evolutionary connection. The genus (if allocated) of phages in these analyses is illustrated.

Analysis with VIPtree allowed the construction of a proteomic phylogram of these phages utilizing 863 genomes. This allowed the placement of these phages in a clade of 14 other related phages possessing genomes ranging from 34 to 52 kbp in size. These related phages possess virions with a siphovirus morphology that infect gram-positive bacterial hosts (Supplementary information 1, Figure S6). Further phylogenetic analysis of this clade using VICTOR to construct a proteomic tree show that the *Ruminococcus* phages closest evolutionary relationship is that placed within the genus of *Svunavirus* (Figure 3a). This observation was further confirmed by Gegenees (tBLASTx), with proteomes of the *Ruminococcus* phages sharing the greatest level of homology (19–20% identity) among inspected phages (Figure 3b). However, the connection between the *Ruminococcus* phages and this phage genus is distant due to the phylogenetic distance indicated in the VICTOR phylogram and low protein homology in Gegenees analysis.

### 3.5. Phage–host interaction in a murine *in vivo* model

**Figure 4.**
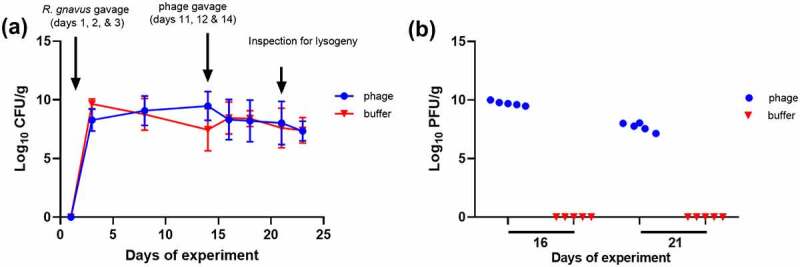
A mouse trial to investigate the interaction of *R. gnavus* JCM 6515^T^ and a *Ruminococcus* phage mixture. (a) CFU/g counts of *R. gnavus* and (b) PFU/g counts of *Ruminococcus* phages in mice faeces from those treated (*n* = 5) and untreated (*n* = 5) with phage. Time points at which mice were gavaged with host bacterium and phages and when *R. gnavus* was inspected for phage lysogeny are illustrated.

A 28-day mouse trial was conducted to assess the dynamics of the six *Ruminococcus* phages and the potential infection of their host within the murine GIT (experimental design presented in Supplementary 1, Figure S7). Germ-free mice were gavaged with *R. gnavus* JCM 6515^T^ at the onset of the trial (days 1, 2 & 3) in two equal groups of mice (*n* = 5). On days 11, 12 and 14, each group of mice were gavaged with either SM buffer or a *Ruminococcus* phage mixture (phiRg507T2/2, phiRg507T2/3, and phiRgPS6). Post-treatment, mice from both groups followed a similar trend of body weight gain till the end of the trial (Supplementary information 1, Figure S8). Post-gavage of *R. gnavus* (from days 3 to 23), the bacterium could be consistently enumerated in mouse feces of both treatment groups, with counts ranging between 3 × 10^5^ and 4 × 10^10^ CFU/g (Figure 4a). No significant difference in bacterial numbers could be detected post phage administration in the phage treatment group. Furthermore, viable phage could be detected on the two inspected time points (days 16 & 21) post-gavage among the phage-treated group, with counts ranging from 1.4 × 10^7^ to 1 × 10^10^ PFU/g, with MOI values ranging between 1 × 10^−2^ to 1 × 10^4^ (Figure 4b). These findings indicate that phages were indeed able to reach the gut of mice and replicate on the *R. gnavus* host without causing a significant reduction of the host population density.

On day 23, a PCR targeting *Ruminococcus* phages was conducted on bacterial isolates (triple streaked to ensure purity) from the phage-treated group. This investigation found that 35 out of 72 (48.6%) colonies tested positive for the presence of prophages phiRgPS6 and phiRg507T2/2 or phiRg507T2/3, indicating their lysogenization by these phages.

### 3.6. Lysogeny of the *Ruminococcus* phages

We were interested in further studying the lysogenization of *R. gnavus* JCM 6515^T^ by the phages isolated in this study to understand how this interaction may impact the ecology of such phages and *R. gnavus* in the gut.

Liquid cultures of *R. gnavus* JCM 6515^T^ lysogenized by phages phiRgPS6 (clones 1–1 and 2–1), phiRg507T2/2 (clone 11) and phiRg507T2/3 (clones 21–1 and 22–1) were inspected for the production of active phage virions resulting from subpopulations of cells where prophages have entered the lytic cycle. Filtered supernatants were spotted onto an overlay seeded with a non-lysognised JCM 6515^T^ where it was possible to detect zones of lysis indicating the presence of viable phage. Indicating that *R. gnavus* lysogenized by these phages possess a sub-population of cells that undergo spontaneous lysis, releasing viable phage in the gut.

We proceeded to determine the range of superinfection immunity conferred to *R. gnavus* JCM 6515^T^ by lysogenization. The sensitivity of *R. gnavus* JCM 6515^T^ lysogenized by either phage phiRgPS6, phiRg507T2/2 or phiRg507T2/3 was investigated by spot assay using lysates of the six *Ruminococcus* phages (Table 2). Phages could not plaque the cultures of JCM 6525^T^ lysogenized with the same phage. Additionally, a crossover of resistance was observed among isolates of JCM 6515^T^ lysogenized by phiRg507T2/2 and phiRg507T2/3, where lysates of either phage could not plaque on these isolates. Additionally, phage phiRg519T2 was found to be unable to plaque on JCM 6525^T^ lysogenized by phiRg507T2/3.

**Table 2.** Sensitivity of *R. gnavus* JCM 6515^T^ with prophage of *Ruminococcus* phages against lysates of the same phages.

Phage	JCM 6515^T^ lysogenised by (isolate)		
	phiRgPS6 (1–1)	phiRg507T2/2 (11)	phiRg507T2/3 (21–1)
phiRgPS6	-	+	+
phiRg507T2/2	+	-	+
phiRg507T2/3	+	-	-
phiRg519T2	+	+	-

Results recorded as −, no plaque formation; +, plaque formation.

**Figure 5.**
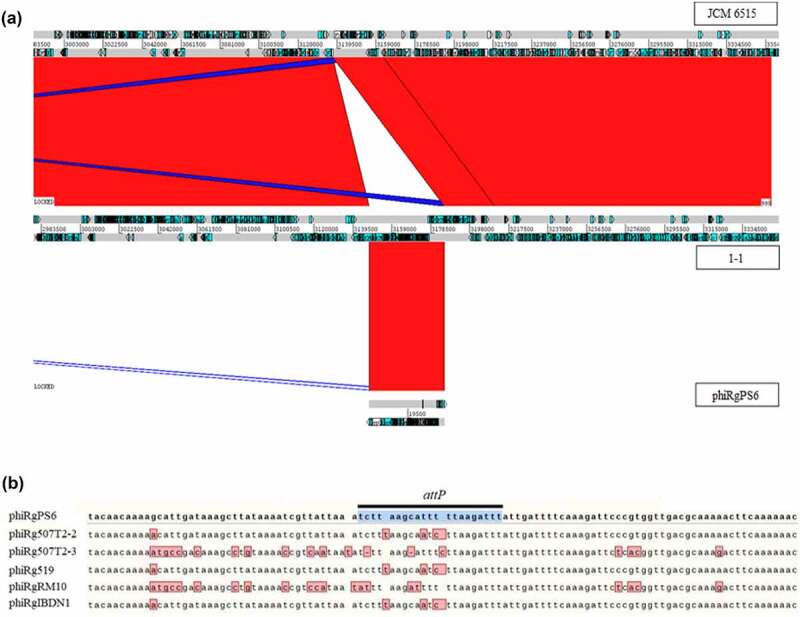
Lysogenetisation of *R. gnavus* JCM 6515T by *Ruminococcus* phage phiRgps6. (a) Genome alignment visualised with the Artemis Comparison Tool (ACT) of the wild-type (WT) *R. gnavus* JCM 6515^T^ versus a clone 1–1 lysogenised by *Ruminococcus* phage phiRgps6, as well as the genome of the phage phiRgps6. Locations of direct homology (as determined with BLASTn) between aligned genomes are indicated with areas of red. (b) Genome alignment of the *attP* of *Ruminococcus* phage phiRgps6 with the other five *Ruminococcus* phages in this study, illustrating sequence diversity of potential *attP* sites among their genomes.

We were also curious to learn how these prophages integrate into the genome of their host. To determine this, cultures of *R. gnavus* JCM 6515^T^ lysogenized by phage phiRgPS6 (clones 1–1 and 2–1) were subjected to short and long read-based sequencing to obtain complete circular genomes (coverage > 100×). Alignment of these genomes to the native wild type JCM 6515^T^ shows that the genome of phage phiRgPS6 had integrated into that of the host. For clone 1–1, an intergenic region between CDSs FXV78_RS15645 and FXV78_RS15640 (position: 3,137,358–3,137,378 bp) was identified as the *attB* site for this phage (Figure 5a). This sequence was identified to be 21 bp in length with the same sequence associated with the *attP* site of phage phiRgPS6 (position: 9,654–19,674 bp, sequence: 5’ – tcttaagcatttttaagattt − 3’). Furthermore, this sequence was found to be preserved with the *attL* and *attR* for the prophage form of phiRgPS6.

A conjugation element is located at the boundary of the prophage integration site (FXV78_RS15645 – FXV78_RS15800) for JCM 6515^T^ clone 2–1, with a similar element found upstream (FXV78_RS12270 – FXV78_RS12425). At the boundaries of these elements, there appears to be the possibility of a genomic inversion that is 668,564 bp in size, as found in clone 2–1 (Supplementary information 1, Figure S9). This observation may relate to the presence of integrase genes situated at the boundary of these conjugation elements.

PCR was conducted on JCM 6515^T^ clones 1–1 and 2–1 utilizing primers targeting the prophage and host genome to confirm the observations made in genomic sequence analysis Supplementary information 2, Table S1. These PCRs resulted in amplicons of the predicted size (Supplementary information 1, Figure S10) that were subsequently sequenced to confirm the location of the prophage.

We also examined publicly available genomes of *R. gnavus*, found to contain prophages sharing similarity at the nucleotide level with the phages of this study. Analysis of the boundary points of these elements allowed identification of those with a predicted *attB* site located at a similar intergenic region (relative to the genome of JCM 6515^T^) as with *R. gnavus* JCM 6515^T^ with a prophage of phiRgPS6 (Supplementary information 2, Table S7). It also appears that related prophages can target *attB* sites associated with tRNA genes (Supplementary information, Table S7). Additionally, the alignment of the *attP* site of phage phiRgPS6 to the other five *Ruminococcus* phages shows sequence diversity at this locus (Figure 5b).

### 3.7. Prevalence of *Ruminococcus* phages in the human gut virome

**Figure 6.**
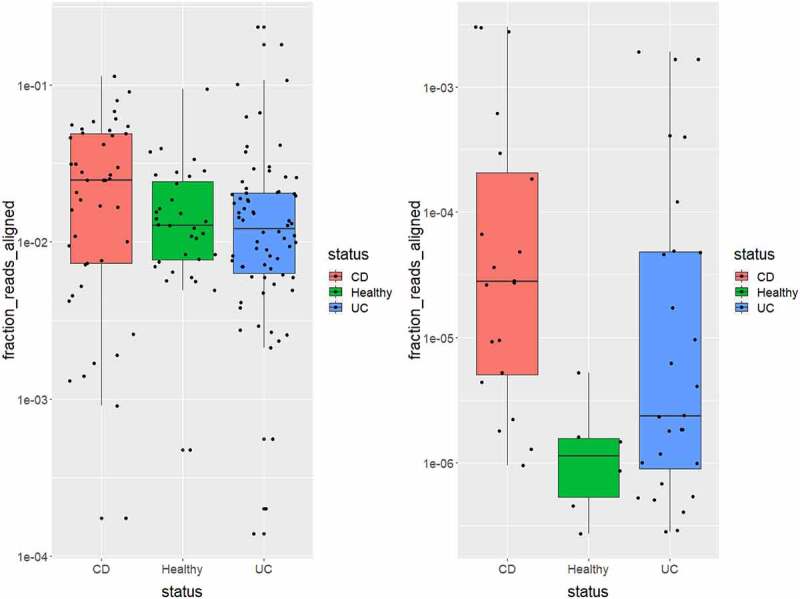
Prevalence of *Ruminococcus gnavus* and *Ruminococcus* phages in examined human faecal 16S rRNA and viriome sequence data. (a) the fraction of reads aligning to *R. gnavus* 16 rRNA gene from faecal 16S rRNA data and (b) the fraction of reads aligned from faecal viromes of individuals with Crohn’s disease (CD), ulcerative colitis (UC) and those deemed healthy with the genomes of the six *Ruminococcus* phages isolated in this study.

As previously discussed, the abundance of *R. gnavus* has been reported to be higher in individuals suffering from IBD than in healthy individuals, especially among those with CD.^15–17^ We were interested in determining if a similar trend could be observed for *Ruminococcus* phages. To determine if that was the case, we reexamined sequence data from the study of IBD and healthy gut microbiomes and viromes reported by Norman *et al*. ^55^ The 16S rRNA dataset from this study is composed of 45 people suffering from CD, 70 people suffering from UC and 32 healthy individuals. Enumeration of reads that aligned (97% identity) to representative 16S rRNA genes of *R. gnavus* shows an increased abundance of the bacterium among individuals with CD compared to the healthy controls (Figure 6a). The virome dataset of the Norman *et al*. study is composed of 37 individuals suffering from CD, 42 individuals with UC, and 64 healthy individuals. We could detect sequence reads aligning to the genomes of the *Ruminococcus* phage among 9% of the healthy individuals, 54% of the individuals with CD and 42% of the individuals with UC. Furthermore, the highest number of aligned reads were detected among viromes of individuals suffering from CD and UC (Figure 6b).

## 4. Discussion

To date, little is known about phages that infect *R. gnavus* and how their infection may affect the behavior of this bacterium in the human gut. In this study, we report the isolation and characterization of six novel phages that infect the type strain of *R. gnavus* JCM 6515^T^. The phages have a siphovirus morphology, with their host range limited to no more than two out of a panel of 10 *R. gnavus* strains, with phages possessing a temperate lifestyle. The phage genomes range between 36.5 and 37.8 kb in size, with genes encoding hallmark proteins implicated in the lysogenic life cycle, such as integrase and putative repressor proteins containing the Cro/C1-type HTH domain (IPR001387).

We demonstrated the lysogenization of *R. gnavus* strain JCM 6515^T^ by *Ruminococcus* phage phiRgPS6, where the *attB* site for this phage is located within an intergenic region on the boundary of a conjugation element. Analysis of public databases allowed the identification of *R. gnavus* genomes with prophage elements that share significant nucleotide similarity (up to 68%) with these newly isolated *Ruminococcus* phages. Furthermore, examining these prophage elements allowed the identification of strains with a predicted *attB* site situated at a similar intergenic region as found for phage phiRgPS6. We also identified similar prophage elements with a predicted *attB* site associated with a tRNA gene. The latter observation has been made among other species of gut bacteria.^61^ The alignment of the known *attP* site of phage phiRgPS6 with the five other *Ruminococus* phages demonstrated a diversity of the nucleotide sequence within this region among the six phages. This diversity may indicate that the six phages target different *attB* sites at different loci that can occur within an *R. gnavus* genome. However, it may also ensure such phages can overcome the emerging sequence diversity of their target *attB* site that is likely to occur among different strains of their host bacterium.

We also show that when *R. gnavus* JCM 6515^T^ is lysogenized by phages phiRgPS6, phiRg507T2/2 and phiRg507T2/3, it becomes immune to lytic superinfection by the same phage. It is possible to observe cross-immunity with cultures of JCM 6515^T^ lysogenized with different prophages. For example, JCM 6525^T^ lysogenized with phiRg507T2/2 was resistant to lysis by phiRg507T2/3. The observed superinfection immunity is expected to be a consequence of prophage repressor proteins simultaneously maintaining a prophage in the lysogenic cycle and interfering with lytic infection by a similar temperate phage.^62^ However, this finding did not explain the lack of plaque formation with the *Runminococcus* phages among the additional nine *R. gnavus* strains used in host range analysis, as these bacterial strains were found not to possess prophage elements with homology to the phages examined in this study.

CRISPR-Cas is an adaptive immune system found among archaea and bacteria that acts against foreign invading DNA. These systems store foreign DNA sequences from past exposures as “spacers” that form part of the CRISPR arrays. Transcribed spacers (short crRNAs) form a complex with Cas proteins that cleave foreign DNA possessing a corresponding protospacer sequence.^63^ We examined the presence of CRISPR arrays among these *R. gnavus* genomes, where they were detected in strains CCUG 51289, CCUG 43437 and JCM 6515^T^ downstream of Cas proteins related to a type I-C system. However, this generated more questions than possible answers for strain JCM 6515^T^ regarding its sensitivity to *Ruminococcus* phage infection. All three strains possess spacers targeting protospacers with no more than three base mismatches found among the six *Ruminococcus* phages. Additionally, JCM 6515^T^ had spacers that perfectly matched protospacers found among phages phiRg507T2/2 and phiRg507T2/3. Their lack of effect against infection by these phages may relate to a mismatch of the PAM sequence associated with protospacers or may suggest that these phages may possess proteins with anti-CRISPR activity.^64,65^ Other avenues that remain to be explored for phage resistance among *R. gnavus* strains include the action of restriction-modification systems, the presence of abortive infection systems and cell surface modifications that could impede phage absorption.^66^

In a mouse trial utilizing germ-free mice mono-colonized with *R. gnavus* JCM 6515^T^ over 27 days, no significant reduction of this bacterium was detected in mouse feces after gavage with a mixture of phages (phiRg507T2/2, phiRg507T2/3 & phiRgPS6), 11 days post gavage with the host bacterium. Additionally, phages could be detected in the feces of mice (days 16 & 21), suggesting phage and the bacterial host could exist in the mouse gut in a manner that did not significantly impact host numbers. There are two mechanisms through which these phages could interact with the host bacterium that could explain this observation. First, phages can infect the bacterium through the lytic life cycle but do so in a manner where an insufficient number of cells are infected or at a speed that does not outpace the growth of the host bacterium (below the inundation threshold).^67^ Second, phages could lysogenize the host, and a subpopulation could release viable phages. The latter option is supported by a phage-specific PCR that confirmed that 48.6% (35/72) colonies of *R. gnavus* JCM 6515^T^ isolated from feces (day 23 of the trial) were lysogenized by phage. Furthermore, liquid cultures of *R. gnavus* JCM 6515^T^ lysogenized by phage phiRgPS6 spontaneously release viable phage. However, the observed outcome of the mouse trial may likely result from *Ruminococus* phages interacting with *R. gnavus* through a combination of both mechanisms.

We also show that *Ruminococcus* phages, like those isolated in this study, are more prevalent and abundant in the gut of individuals with IBD, as determined by the alignment of publicly available gut virome sequence reads from 143 individuals. The prevalence and abundance of these phages among individuals with IBD, especially CD, correlate with the high prevalence and abundance of *R. gnavus* typically found among such individuals.^15,17,68^

Our results indicate that there is more likely a positive rather than a negative correlation between the high prevalence and abundance of *R. gnavus* within the gut of those with IBD and the type of *Ruminococcus* phages isolated in this study. This conclusion begets some interesting questions that merit further investigation. For example, if a high number of such phages can occur alongside a high number of *R. gnavus*, does this result in increased lysis events of the host bacterium within the gut? If so, can the products of *R. gnavus* lysis play a role in stimulating the human immune system that induces inflammation associated with IBD? Indeed, lytic phage infection has been shown to release cellular components (Pathogen Associated Molecular Patterns) that can elicit an immune response.^69^ Follow-on work that may help answer these questions could include *in vitro* assays based on immune system cells to examine pro-inflammatory signals.^19^ Or animal trials using interleukin-10-deficient mice that are inoculated with *R. gnavus* with the phages of this study versus the bacterium alone, allowing the detection of an altered immune response.^70^

## Supplementary Material

Supplemental Material

## Data Availability

The genomes of bacteria used are freely available on the NCBI website, accession numbers are provided in Table 1.
