# Supplementary material for: Temperate bacteriophages infecting the mucin-degrading bacterium Ruminococcus gnavus from the human gut
Source: Gut Microbes. 2023 Mar 30;15(1):2194794. doi: 10.1080/19490976.2023.2194794 (PMC10072058; doi:10.1080/19490976.2023.2194794)
Supplement: Supplemental Material [file KGMI_A_2194794_SM5769.zip › Supplementary material/Supplementary_information_1_FINAL.docx]

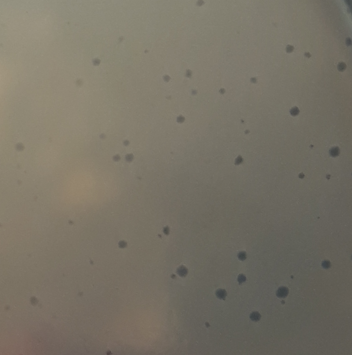


**Figure S1**. Halo zones surround *Ruminococcus* phage (left) Rg507T2/3 plaques (left) Rg507T2/2 (right).

**Figure S2**. Heatmap showing nucleotide similarity of *Ruminococcus* phages as calculated with VIRIDIC


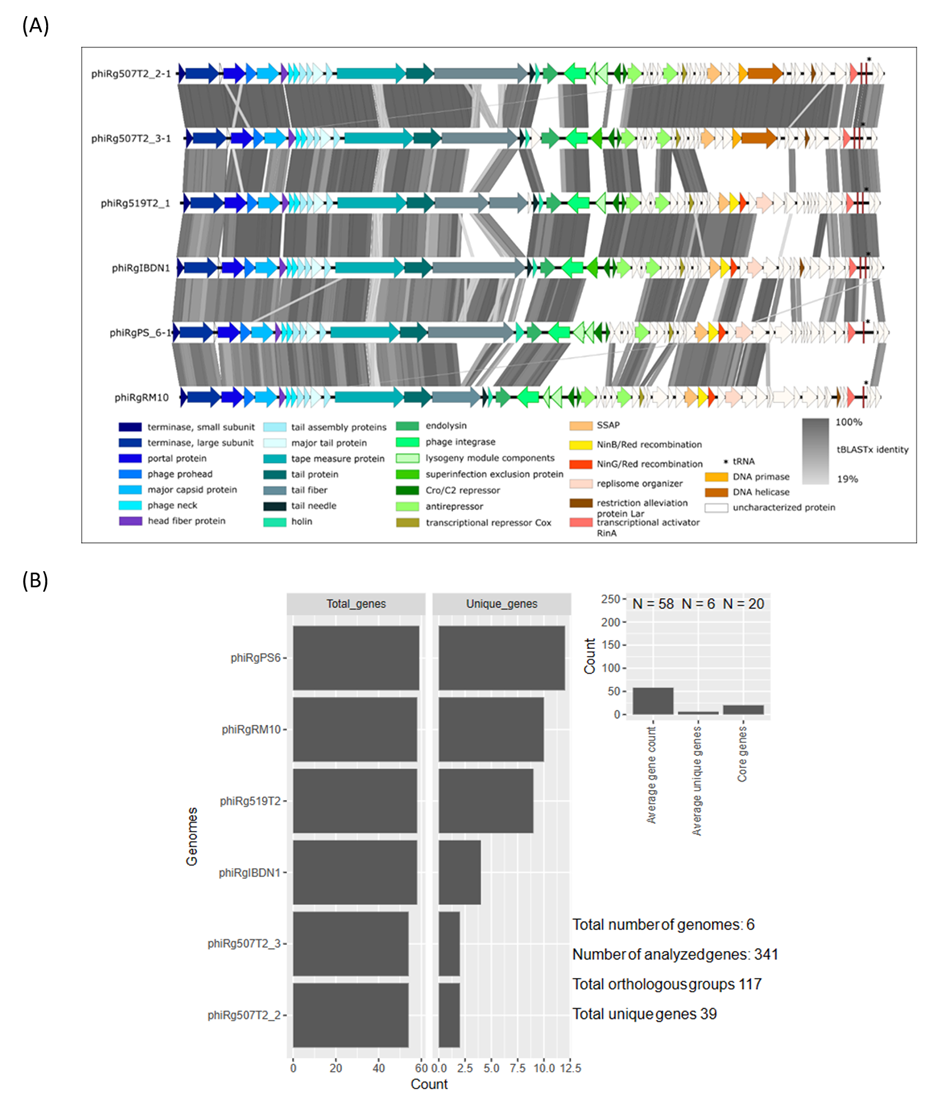


**Figure S3**. Numerical summary of the pangenome analysis using Proteinortho (Identity=30%, Coverage = 70%) of the six *Ruminococcus* phages.


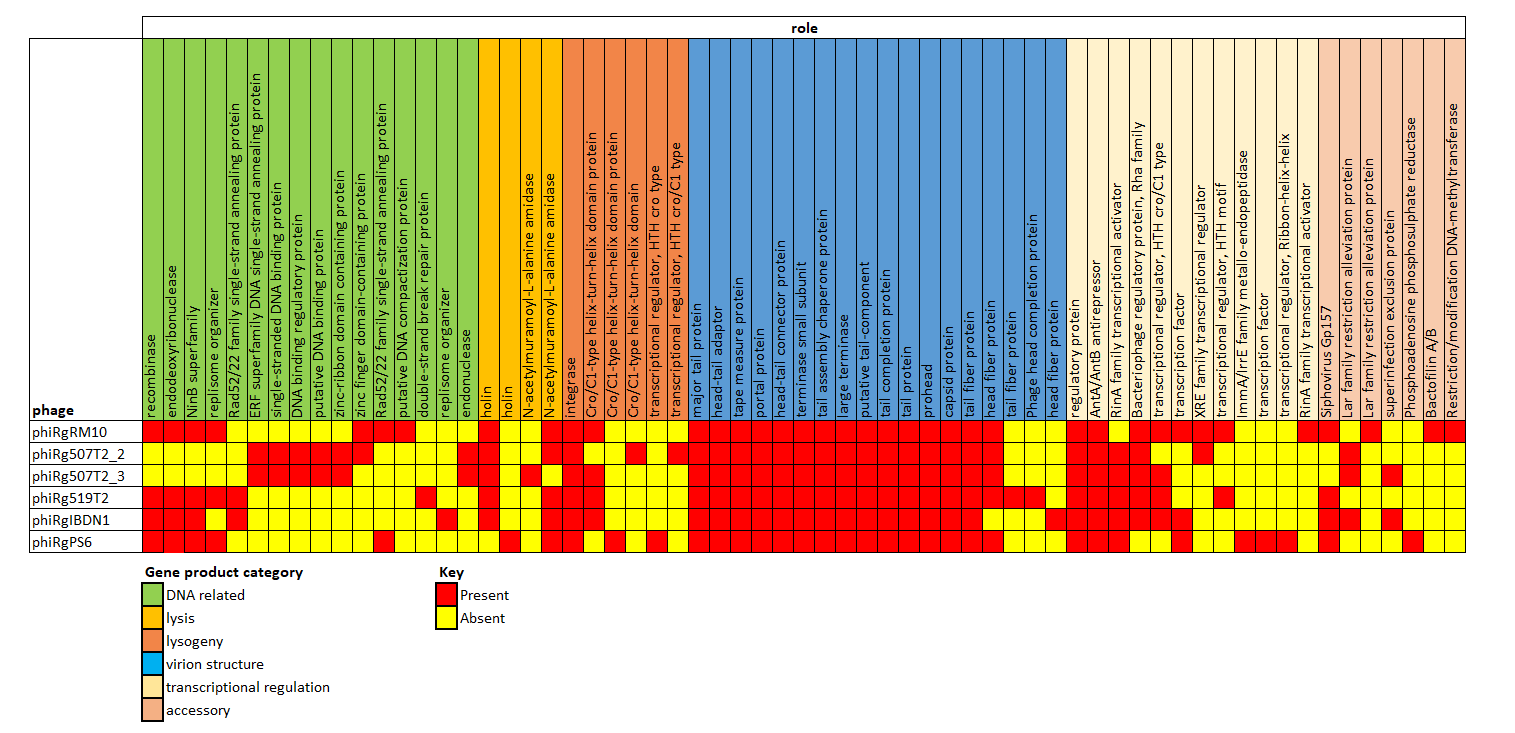


**Figure S4.** Heatmap illustrating genes detected among *Ruminococcus* phages found with a particular function. Heatmap illustrates shared genes as calculated by Proteinortho (Identity = 30%, coverage = 70%) among *Ruminococcus* phages where function of their gene product could be determined. Key describes colour coding for the presence absence of genes among phages and the category gene product function is allocated for the gene product in question.


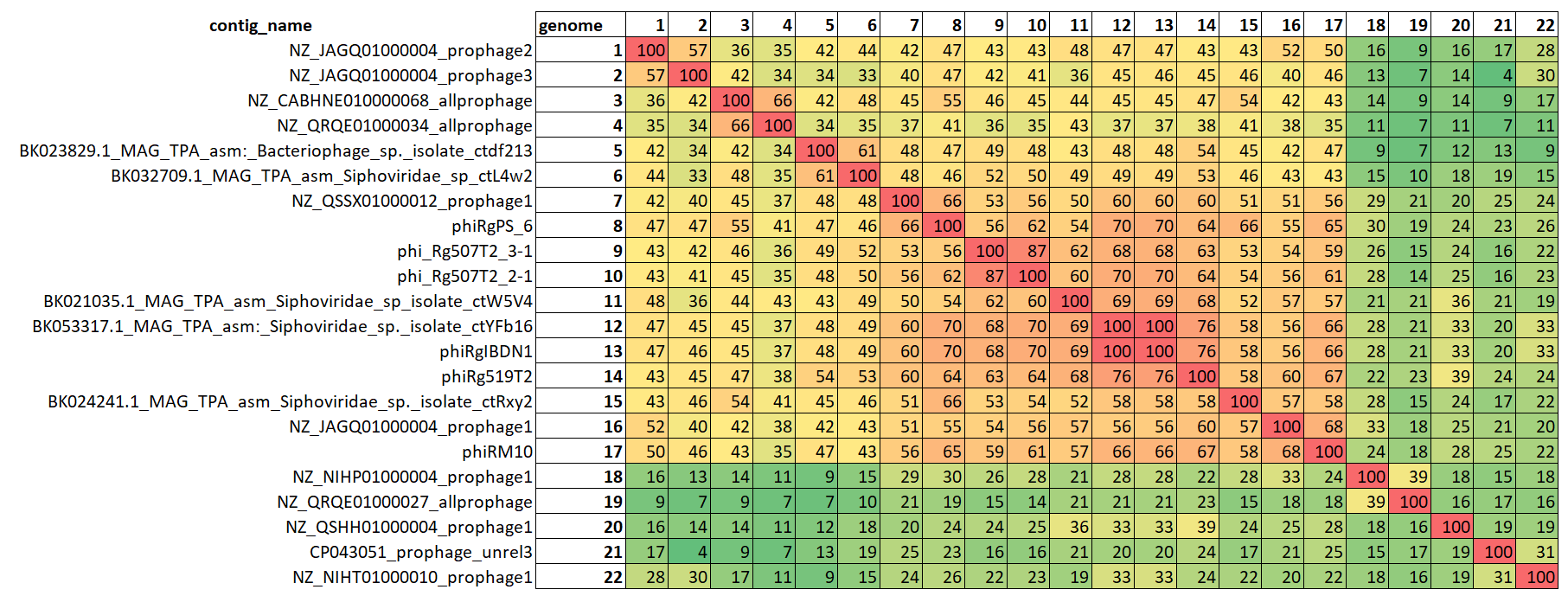


**Figure S5**. Heatmap showing nucleotide similarity of *Ruminococcus* phages isolated in this study and MAGs obtained from human microbiomes and prophage elements extracted from genomes of *Ruminococcus gnavus* as calculated with VIRIDIC.


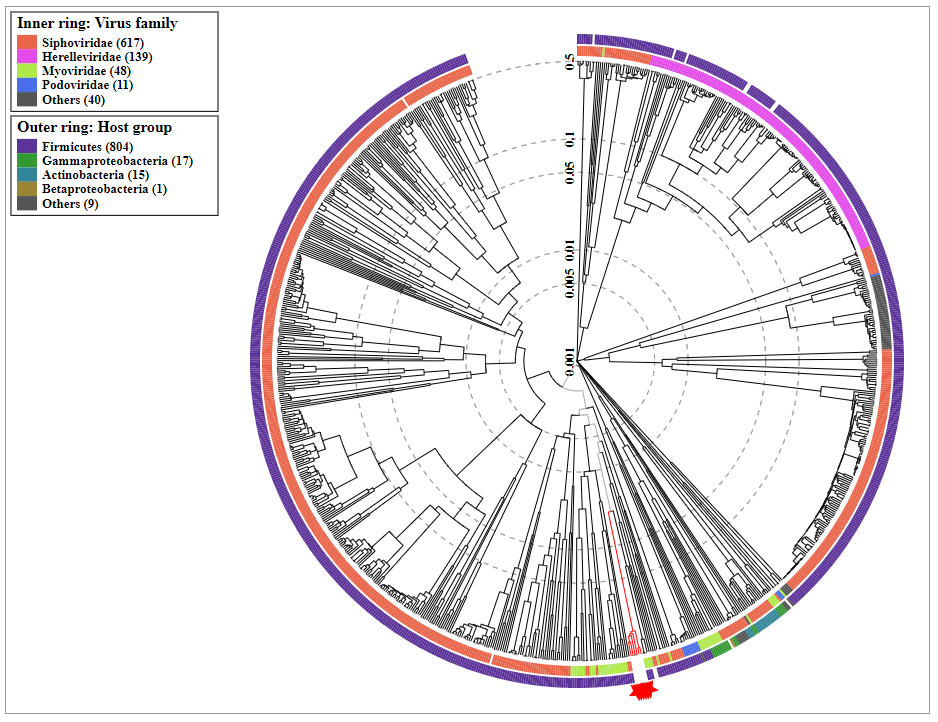


**Figure S6**. A circular proteomic phylogram plotted by VIPTree using phage genomes of 863 genome sequences including six *Ruminococcus* phages of this study. Position of the six *Ruminococcus* phages on the phylogram are highlighted with red stars.


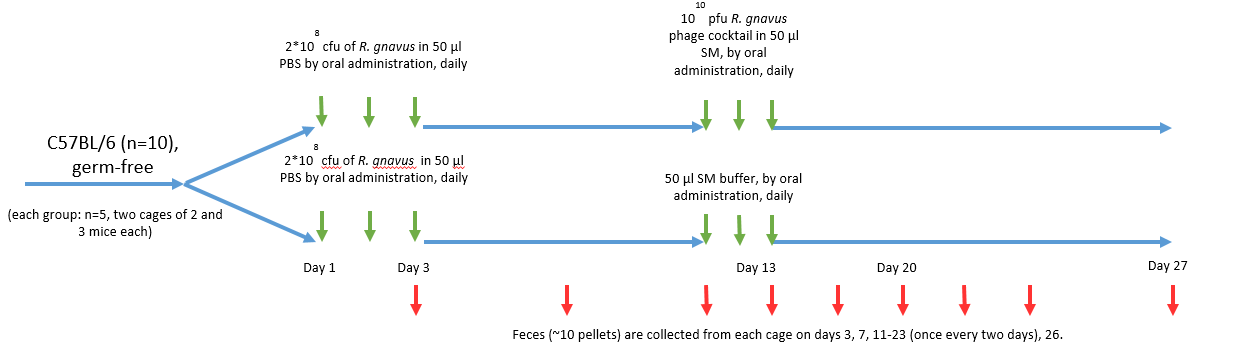


**Figure S7.** Diagram illustrating the experimental setup of the mouse trail of this study examining the *Ruminococcus* phages infection of the with their host within the GIT.


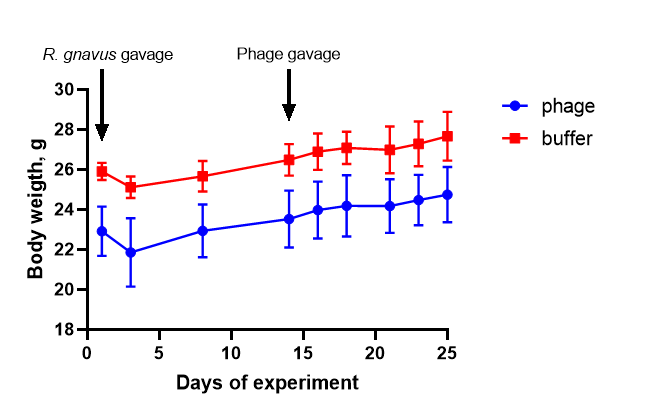


**Figure S8.** The average weight of mice throughout the mouse trail inspecting interaction between *R. gnavus* and *Ruminococcus* phage mixture in an *in vivo* setting.


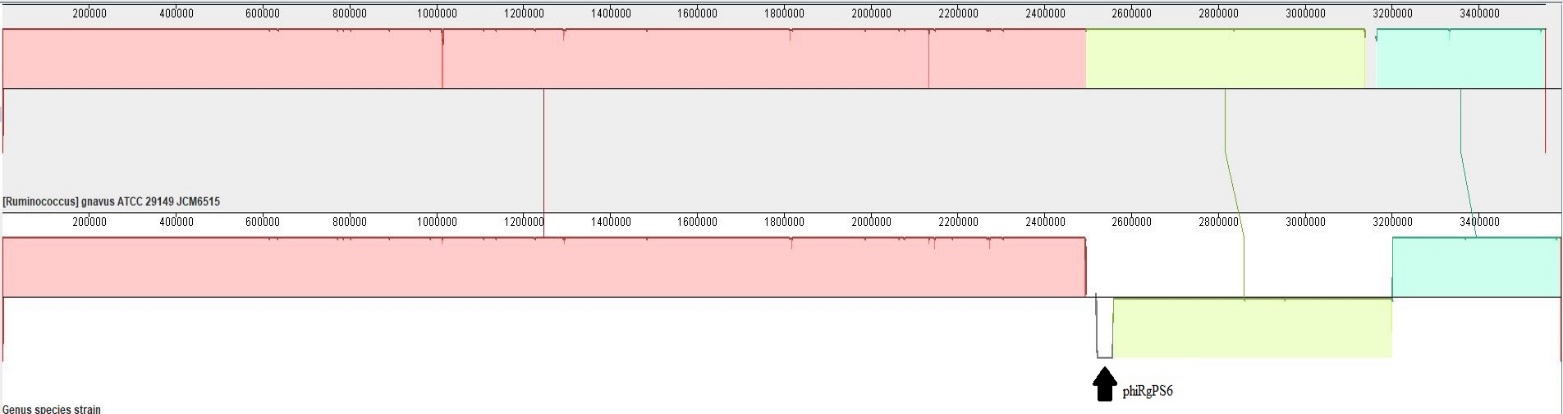


**Figure S9.** Genome alignment visualised with the Mauve of the wild-type (WT) *R. gnavus* JCM 6515^T^, an isolate of bacterium (clone 2-1) lysogenized by *Ruminococcus* phage phiRgPS6. Each genome is laid out horizontally with homologous segments (locally collinear blocks [LCBs]) outlined as coloured rectangles. Regions inverted relative to JCM 6515^T^ are set below those that match in the forward orientation. Lines collate aligned segments between genomes. Sequence similarities within an LCB are proportional to the heights of interior-coloured bars. Location of the genome of phiRgPS6 in that of clone 2-1 is indicated by a black arrow.


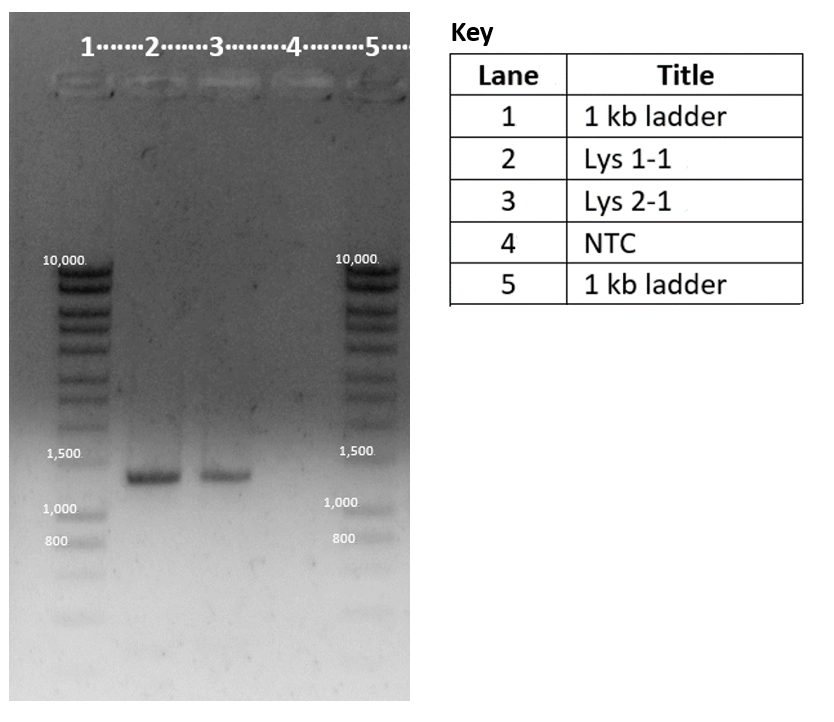


**Figure S10.** Electrophoresis gel image of amplicons resulting from PCRs targeting *Ruminococcus* phage phiRgPS6 integrated into the genome of two isolates (clones 1-1 and 2-1) of *R. gnavus* JCM6515^T^. Key indicated locations of DNA ladder (1 kb Bioline) and PCR amplicons on gel image.
